# Case Report: Precision genetic diagnosis in a case of Dyggve-Melchior-Clausen syndrome reveals paternal isodisomy and heterodisomy of chromosome 18 with imprinting clinical implications

**DOI:** 10.3389/fgene.2022.1005573

**Published:** 2022-11-18

**Authors:** María-Pilar López-Garrido, María-Carmen Carrascosa-Romero, Minerva Montero-Hernández, Caridad-María Serrano-Martínez, Francisco Sánchez-Sánchez

**Affiliations:** ^1^ Laboratorio de Genética Médica, Instituto de Investigación en Discapacidades Neurológicas (IDINE), Facultad de Medicina de Ciudad Real, Universidad de Castilla-La Mancha (UCLM), Albacete, Spain; ^2^ Servicio de Neuropediatría, Complejo Hospitalario Universitario de Albacete, Albacete, Spain; ^3^ Laboratorio de Genética Médica, Instituto de Investigación en Discapacidades Neurológicas (IDINE), Facultad de Medicina de Albacete, Universidad de Castilla-La Mancha (UCLM), Spain

**Keywords:** UPD chromosome 18, DMC syndrome, precision medicine, imprinting chromosome 18, diagnostic odyssey, ELOA3, DYM gene mutation, TCEB3C

## Abstract

A twelve-year-old patient with a previous clinical diagnosis of spondylocostal skeletal dysplasia and moderate intellectual disability was genetically analyzed through next generation sequencing of a targeted gene panel of 179 genes associated to skeletal dysplasia and mucopolysaccharidosis in order to stablish a precision diagnosis. A homozygous nonsense [c.62C>G; p.(Ser21Ter)] mutation in *DYM* gene was identified in the patient. Null mutations in *DYM* have been associated to Dyggve-Melchior-Clausen syndrome, which is a rare autosomal-recessive disorder characterized by skeletal dysplasia and mental retardation, compatible with the patient´s phenotype. To confirm the pathogenicity of this mutation, a segregation analysis was carried out, revealing that the mutation p(Ser21Ter) was solely inherited from the father, who is a carrier of the mutation, while the mother does not carry the mutation. With the suspicion that a paternal disomy could be causing the disease, a series of microsatellite markers in chromosome 18, where the *DYM* gene is harbored, was analyzed in all the members of the family. Haplotype analysis provided strong evidence of paternal isodisomy and heterodisomy in that chromosome, confirming the pathological effect of this mutation. Furthermore, the patient may have a compromised expression of the *ELOA3* gene due to modifications in the genomic imprinting that may potentially increase the risk of digestive cancer. All these results highlight the importance of obtaining a precision diagnosis in rare diseases.

## Introduction

Dyggve-Melchior-Clausen (DMC) syndrome [MIM# 223800] is a rare disease included in the heterogenous group of spondylo-epi-metaphysal dysplasias disorders, all of them defined by the combination of vertebral, epiphyseal and metaphyseal abnormalities. DMC syndrome is characterized by short trunk dwarfism, microcephaly and mental retardation (Beighton, 1990). The prevalence of this syndrome is < 1/1,000,000, and there are about 100 cases reported worldwide (Orphanet, 2022).

DMC syndrome is caused by homozygous null mutations in the *DYM* gene (Dimitrov et al., 2009; Dupuis et al., 2015). This gene (NCBI ID: 54,808) is located in chromosome 18 (18q21.1) (Cohn et al., 2003; el Ghouzzi et al., 2003) and is consists of 17 exons which encode dymeclin, a 669-amino acid protein whose function remains unknown. However, it has been proposed that it may be an integral protein of the endoplasmic reticulum membrane that could play a role in the transport of intracellular compounds. Furthermore, it could also have a critical role in both the formation and function of the Golgi apparatus and in the tracking of associated vesicles.

Dymeclin is highly conserved across species, but it does not belong to any identified protein family. This protein is expressed at various embryonic stages of human brain development, chondrocytes, osteoblasts and skin fibroblasts (el Ghouzzi et al., 2003).

Uniparental disomy (UPD) refers to the inheritance of both copies of a chromosome from one single parent. This phenomenon usually occurs due to aberrations during meiosis, and two main subgroups can be distinguished: uniparental isodisomy (UPiD), where both allele copies are identical (they originated from the sister chromatids); and uniparental heterodisomy (UPhD), where the pair of homologous chromosomes of a parent is inherited. Recombination processes during meiosis can result in partial UPiD, partial UPhD or mixed UPiD-UPhD (Benn, 2021). The overall prevalence of UPD in the general population is estimated to be 1:2,000 births, whereas the prevalence of UPD as a cause of rare diseases ranges from 1:3,500 to 1:5,000 (Robinson, 2000; Liehr, 2022). To date, only one case of rare disease, concretely hearing loss, have been reported to be caused by a mutation in *LOXHD1* gene and UPD of the chromosome 18 (Morgan et al., 2018).

On the other hand, imprinting is a phenomenon that occurs in some developmental genes and consists in the silencing of one of the copies of the gene (maternal or paternal), thus having a monoallelic expression under normal conditions. Genes with imprinting have been described in all human chromosomes (Geneimprint, 2022). When UPiD occurs in a region with imprinted genes, the expression of those can vary drastically and cause diseases such as the well-known Prader-Willi syndrome, Angelman syndrome or Silver-Russell syndrome, among others (Liehr, 2022). To date, only three imprinted genes (*TCEB3C/ELOA3*, *ZNF396* and *PARD6G*) have been described in chromosome 18 (Geneimprint, 2022).

The case reported here describes the precision diagnosis performed in a patient with DMC syndrome in which the mutation p.(Ser21Ter) was identified in the *DYM* gene, a gene associated with this pathology. The genetic analysis of this result confirms the pathogenic nature of the mutation and reveals that the homozygous state of the mutation was caused by a paternal mixed UPiD-UPhD in chromosome 18, a mechanism that had not been described in this chromosome until now. In addition, the risk of digestive cancer could be particularly increased in this patient due to modifications of the paternal imprinting in the isodisomic region, specifically where a tumor suppressor gene (*ELOA3*) is located.

## Case description

The subject was evaluated at the *Complejo Hospitalario Universitario de Albacete* (Spain).

### Family history

First child of 23-year-old, healthy and non-consanguineous parents.

### Personal history

#### Prenatal period

The pregnancy had a normal course until 33+2 weeks when preeclampsia was detected and corticosteroids were used to promote lung maturation in the fetus. Delivery occurred at 33+5 weeks by cesarean section with a cephalic presentation, clear amniotic fluid (Apgar score: 7/8) and a constitutional hypotony in relation to the prematurity.

#### Neonatal period

The patient was admitted to Neonatology Service due to prematurity. The examination was normal, without dysmorphias and with parameters in accordance with prematurity. On the second day of life, jaundice was detected with a maximum bilirubin level of 12.6 mg/dl. The patient required phototherapy for 72 h and was discharged after 20 days of life with no other incidents. Before the patient was 1 year old, the family moved to their country of origin (Ecuador).

#### Infant period

At 3 years old, the parents detected a significant developmental delay (free walking at 20 months and language delay limited to referential disyllables) and decided to return to Spain. Psychological examination revealed hyperactivity and an attention deficit disorder. Physical examination showed a weight of 9,600 g (<p3), a cranial perimeter of 46 cm (<p3), a height of 84 cm (<p3), short trunk and neck dwarfism. At that moment, the patient was diagnosed with microcephaly, kyphosis, peptum carinatum, flat feet and a spondylo-epi-metaphyseal dysplasia. Other complementary examinations were performed such as karyotype, hemogram and biochemistry, that were normal. The metabolomic study also detected normal levels of amino acids, organic acids, and sialotransferrins. Lysosomal diseases such as the Morquio syndrome were discarded due to the absence of corneal clouding, deafness, mucopolysaccharidosis or valvular disease. Pathologic skeletal X-rays showed generalized platyspondyly of the whole vertebral rachis, bilateral coxofemoral dysplasia, and metaphyseepiphyseal widening of long bones. According to this evidence, a Spondyloepiphyseal Dysplasia diagnosis was made. However, the patient did not yet have a precision diagnosis. Multidisciplinary follow-up was carried out in the Dysmorphology/Neuropediatrics, Endocrinology and Pediatric Orthopedics Services up to 9 years old. Two years later, he underwent surgery for flat feet and two subtalar implants were inserted in both feet. But it is not until the child is 12 years old that the patient returns to the Dysmorphology/Neuropediatrics consultation. The child had the same clinical characteristics that had been previously detected, but, in addition, he was diagnosed with moderate intellectual disability. At that time, a NGS panel of 179 genes associated with skeletal dysplasia and mucopolysaccharidosis was requested by the doctor.

The NGS company in charge of the sequencing reported a homozygous mutation in the *DYM* gene and classified it as a variant of uncertain significance. This variant was previously found in heterozygous state in a single Asian individual (frequency of 4 × 10^−6^) according gnomAD data base. A family segregation study was recommended by the company to confirm the pathogenic nature of the mutation.

**FIGURE 1:**
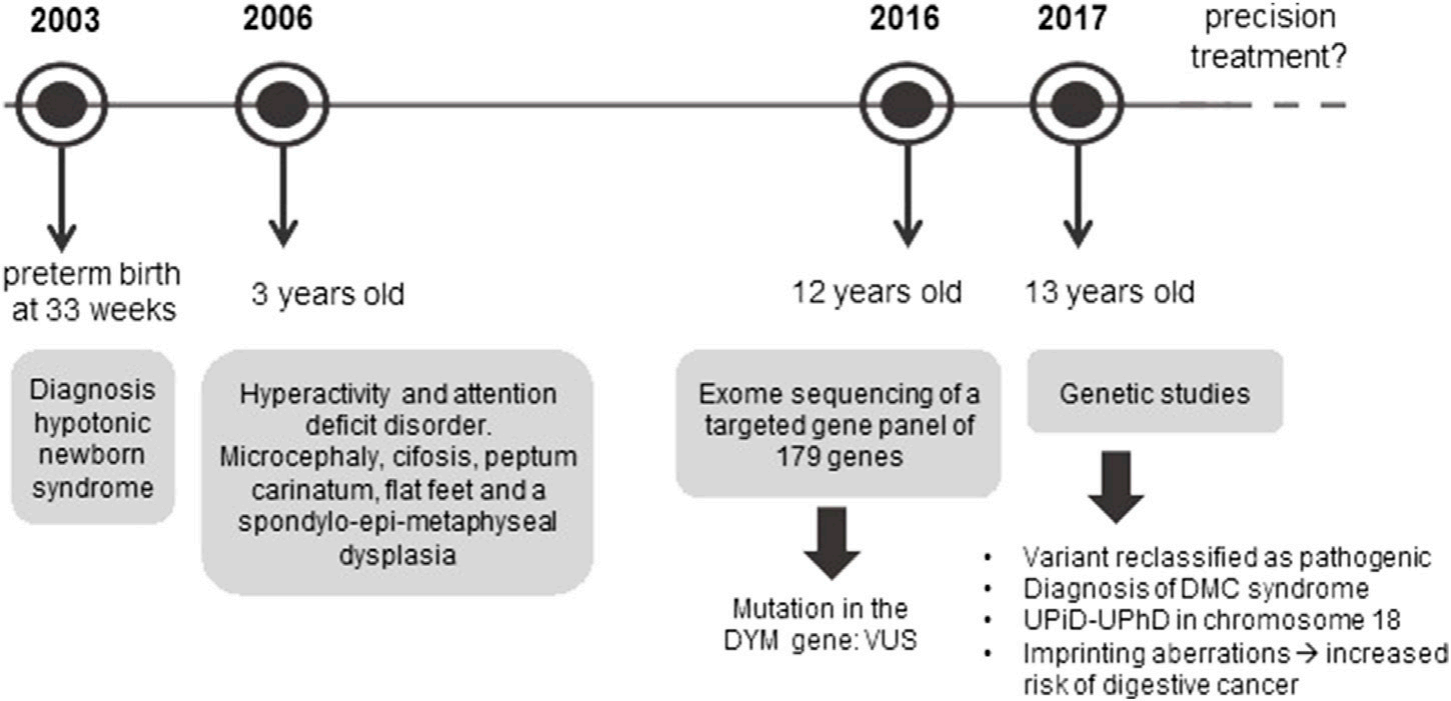
Diagnostic odyssey timeline of the patient. The figure shows the different stages that this patient has undergone since the appearance of the first symptoms until a precise diagnosis has been achieved. Once the patient is given a diagnosis, the main challenge is to develop a precision treatment.

In light of these results, in 2017 the case was referred to our Medical Genetics laboratory, where the mutation was analyzed, and other genetic studies were performed so as to offer a precision diagnosis and genetic counselling to the family. Thus, the variant was reclassified as pathogenic and DMC syndrome was confirmed. At the same time, the UPiD-UPhD and the genetic mechanism responsible for the disease in this patient, as well as the clinical consequences of this condition, were reported to the Dysmorphology/Neuropediatrics Service of the *Complejo Hospitalario Universitario de Albacete*, putting an end to 13 years of diagnostic odyssey (Figure 1).

## Subjects and methods

### Subjects

Four family members (parents and their two sons) were recruited in the Department of Pediatrics of the “Complejo Hospitalario Universitario de Albacete”, Spain, for genetic analysis. The study followed the tenets of the Declaration of Helsinki and informed consents were obtained from the parents. The patient and his family were clinically evaluated by experienced neuropediatricians. Except for the patient, the rest of the family members did not report any pathology of interest at the time of the study.

### Genetic analysis

DNA extraction: Genomic DNA was obtained from peripheral blood leukocytes in all family members using the E.Z.N.A. Blood DNA kit (Omega Bio-tek) according to manufacturer’s protocol.

NGS technology: a whole exome sequencing (WES) was carried out and subsequently, a panel of 179 genes associated with skeletal dysplasia and mucopolysaccharidosis was filtered in the case reported by an external company using an Ion Proton (Life Technologies) platform (Supplementary Table S1). The exome capture was designed using the Ion AmpliSeq™ Exome technology (Life Technologies). The analysis was addressed to the identification of variants located in exonic regions or in splice-site junctions that involve a protein modification (nonsense/missense mutations, as well as nucleotide insertions, deletions or indels) with a frequency higher than 40% of the reads.

Sequencing data were aligned to the reference genome (hg19) using the TMAP-Ion-Aligment software (Life Technologies). Variants were further annotated and analyzed using the latest available version of ION Reporter Software (Life Technologies). 99% of targeted sequences were reliably sequenced, with at least 10X coverage.

Filtering out of variants was carried out following these criteria: 1) variants with a high frequency in population (Minor Allele Frequency (MAF) > 1%), and 2) variants detected in genes associated with clinical profiles not related with the observed in our patient were not prioritized to be disease-causing. Applying the first filtering, we eliminated single nucleotide polymorphisms (SNPs) and common variants that have been previously described as polymorphisms in population databases and no association with any clinical phenotype has been reported in the literature. Applying the second criteria, three missense mutations with a low allelic frequency (<0.01; gnomAD) present in three different genes (*SLC34A3*, *TRPV4* and *CTSK*) were eliminated due to the lack of genotype-phenotype correlation in the patient.

Segregation analysis: p.(Ser21Ter) mutation was confirmed in the patient and analysed in the rest of the family members by PCR and Sanger sequencing. The following PCR primers were designed in the intronic regions flanking exon 2 of the *DYM* gene (transcript version ENST00000269445.10; DYM-201):

FW-5′GTTGGGATTACAGGCGTGAG3’; RV-5′TTCCAGAACGGGTCATTCTC3’.

Terminator cycle sequencing was carried out using the BigDye kit (version 3.1; *Applied Biosystems*) and the products of sequencing reactions were analysed in a 3730xl automated DNA analyser (*Applied Biosystems*). Chromas Pro software was used for sequence analysis.

### Haplotype analysis

Seventeen microsatellite markers were evaluated in all family members’ chromosome 18. Markers were amplified by PCR using the primers and conditions detailed in Table 1.

**TABLE 1:** Primer sequences and annealing temperatures used to amplify microsatellite markers of chromosome 18.

Marker	Primer sequences (5' → 3′)		Annealing temperature (°C)
	Forward	Reverse	
D18S59	*H*-AGCTTCTATCCAACAGGGGC	ACC​AGA​ATG​TGA​ACG​ACC​CTA​GG	63
D18S452	*F*-TGGGGCATACATAGTGCAAA	CCTTTTGCTAGTTGGGT	57
D18S1133	*H*-CCCCCACTATACCAGGAGAT	CCA​GTT​GCT​CCA​ACA​AAA​A	*57
D18S57	*H*-TTCAGGGTCTTTTGAAGAGG	AGA​AGG​CAT​TAA​ATT​TTG​CA	57
D18S1136	*F*-CCAAGTTAGTGGGTCTTGTTC	CTT​TTT​GGT​CTT​AGG​TAA​ATT​GTC​T	57
D18S1127	*F*-AGACCCTGGAGAGTGACTGC	TGC​CCA​TGA​ACT​TAG​TGT​GA	57
D18S69	*H*-CATTAGCAGTCTGGAAATCCTC	CGC​TAT​TGT​ACT​GAA​AAC​CTG​A	59
D18S39	*F*-TCAATGAAGTTCTGCATGCT	GTT​CAT​GCT​CTC​ATA​GGA​AC	62
D18S858	*H*-AGCTGGAGAGGGATAGCATT	TGC​ATT​GCA​TGA​AAG​TAG​GA	59
D18S1144	*F*-CTGGATTAGCCAGGCCC	TGA​CTT​GTG​GAC​ACA​TCA​CTC	62
D18S1129	*F*-GGCTGCACAGGCATTC	AGT​CTT​CCA​GGA​CGA​GAC​ATC	62
D18S1103	*F*-GAATCTCTTGAACCAGGGA	AAC​CAG​TAG​GCA​TTT​GGA​A	57
D18S465	*H*-ATATTCCCCTATGGAAGTACAG	AAA​GTT​AAT​TTT​CAG​GCA​CTC​T	*57
D18S483	*H*-TTCTGCACAATTTCAATAGATTC	GAA​CTG​AGC​AAA​CGA​GTA​TGA	57
D18S488	*F*-TTCTGAAGACAGATCCAAGTG	ATC​ATG​TGA​GCC​AAT​TCC​T	57
D18S1161	*F*-GTCCGTCCAACGTCCAA	GGA​GAG​CCA​CAC​CTA​TCC​TG	55
D18S1141	*H*-TCTTTTGACAAATAACCCC	GGACAGTGCGAGACCT	57

*H*: Primer sequence marked with HEX fluorescent dye (green) in 5′ position. *F*: Primer sequence marked with FAM fluorescent dye (blue) in 5′ position. *: PCR reaction medium contains 5% DMSO.

After amplification, 100 ng of PCR product was mixed with 30 µL of formamide and 0.3 µL of GENESCAN^®^ 400HD size standard (*Applied Biosystems*). Fragment analyses were carried out in a 3730xl DNA sequencer (*Applied Biosystems*) and Peak Scanner v1.0 software was used to visualize fragment lengths.

## Results

**FIGURE 2:**
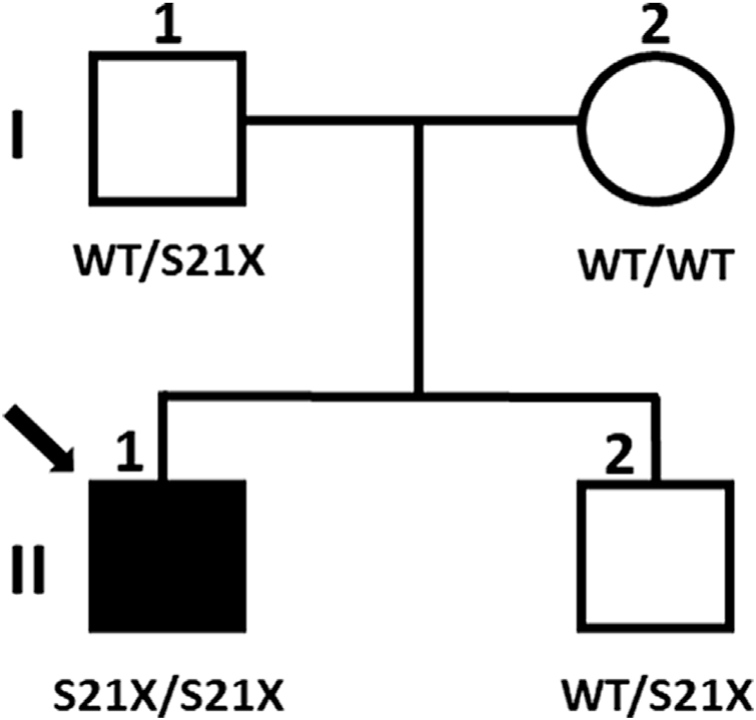
Segregation of the mutation in the family. Filled symbols represent affected individuals, and the arrow indicates the proband subject of this study (individual II:1). Genotypes are indicated below the symbols.

Next generation sequencing company identified a variant of uncertain significance in the patient, NG_009239.2(NM_017653.6):c.62C>G, found in homozygous state in the exon 2 of the *DYM* gene. This change predicts the nonsense mutation NP_060123.3:p.(Ser21Ter) in the dymeclin protein. Although null *DYM* mutations cause DMC syndrome, whose phenotype is compatible with the clinical features of the patient, it was essential to confirm the pathogenicity of the mutation, as it had not been previously described. Therefore, a segregation study was carried out in all family members (Figure 2). The results revealed that the patient inherited the mutation only from his father, who is a carrier of the mutation, but not from his mother, suggesting a possible paternal UPiD. In such a scenario, the segregation pattern would still be consistent with the pathological condition of the mutation, but to achieve a precision diagnosis in the child it was necessary to clarify the molecular mechanism underlying the disease.

**FIGURE 3:**
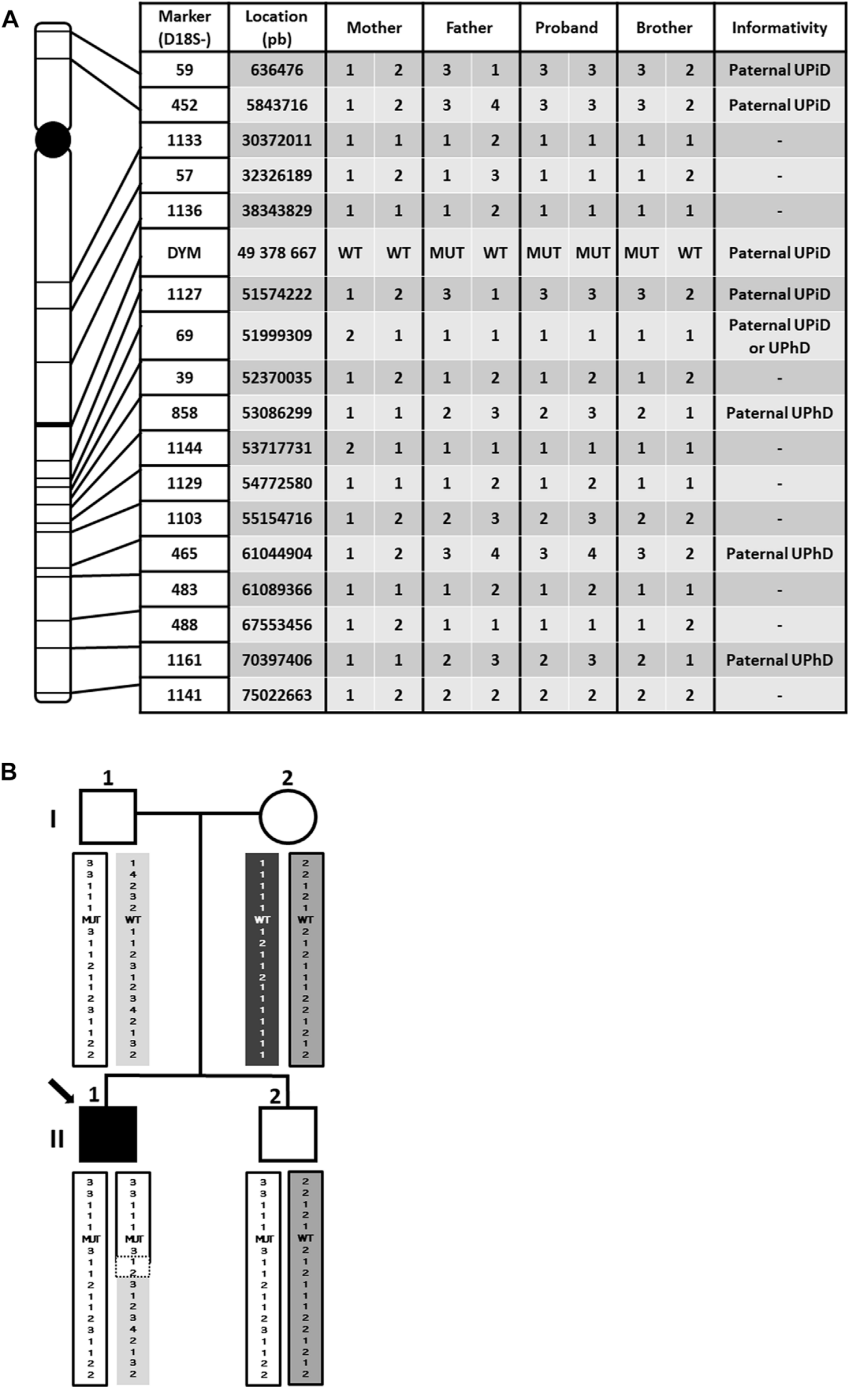
Haplotype genotyping for 17 microsatellite markers. **(A)** Location of markers covering chromosome 18 and haplotype results. UPiD: uniparental isodisomy; UPhD: uniparental heterodisomy. **(B)** Haplotype segregation in the family. The different haplotypes are visualized by color coding. Note that the proband contains a non-informative region delimited by dotted lines, which makes it imposible to establish the cut-off point between UPiD and UPhD. UPiD: uniparental isodisomy; UPhD: uniparental heterodisomy; WT: wildtype genotype; MUT: mutant genotype.

Paternal isodisomy is usually originated by an abnormal chromosomal segregation during meiosis. To confirm this hypothesis, a haplotype analysis with a total of 17 microsatellite markers located throughout chromosome 18 was carried out in all family members (Figure 3).

The results showed that the proband does not share any of the haplotypes of his mother but has the same as those of his father, proving the UPD. However, UPiD of chromosome 18 is not complete, spreading from the distal p-end (short arm) to approximately 51.574.222 pb position (18q21.32), long after the centromere, while both paternal homologous chromosomes (UPhD) spread approximately from this last position to the final downstream location (distal q-end arm). In addition, no small peaks from the mother were detected in the microsatellite analysis performed in the patient, assuming no mosaicism. Therefore, we can state that both UPiD and UPhD coexist in the patient´s chromosome 18. As expected, *DYM* gene is located in the isodisomic region, giving a molecular explanation for the homozygosity of the mutation in the proband.

With these findings and in accordance with the American College of Medical Genetics and Genomics (ACMG) recommendations on the classification of genetic variants (Richards et al., 2015), the mutation was reclassified from variant of uncertain significance (results provided by the NGS sequencing company) to pathogenic with a very strong weighting (PVS1 code in the ACMG classification) as it is a homozygous predicted null mutation originated by a premature stop codon. This loss of function is the mechanism by which the DMC syndrome is developed, and the segregation pattern (with the isodisomy in the *DYM* region) is also compatible with the disease.

## Discussion

In recent years, NGS technology is allowing precision genetic diagnosis to become one of the fundamental pillars on which pediatricians rely when diagnosing rare diseases. Since the frequency of these diseases is low, clinicians are not used to seeing many of these diseases in their practices, so precision genetic diagnosis is of vital importance. DMC syndrome was usually diagnosed by radiological evidence but complementing the diagnosis with genetic analysis can not only reveal the molecular cause and inheritance pattern of the disease, but also provide relevant information for prognosis, treatment and clinical management of the patient.

### Pathogenicity of the mutation in the DYM gene

A panel of 179 genes associated to skeletal dysplasia and mucopolysaccharidosis in a patient with a clinical diagnosis of spondylocostal skeletal dysplasia and moderate intellectual disability revealed a rare mutation, [c.62C>G; p.(Ser21Ter)], in the *DYM* gene. In the present study, it is determined for the first time that this mutation causes DMC syndrome in the proband and that it is caused by a paternal isodisomy-heterodisomy of chromosome 18. This variant predicts a premature stop codon in exon 2 of the 17 exons of the *DYM* gene. Under these circumstances, and with no alternative splicing or second translation initiations beyond exon 2 having been described, the nonsense-mediated mRNA decay (NMD) mechanism would presumably act to eliminate the transcript, resulting in a complete absence of the dymeclin protein. The action of the NMD system in this gene is supported by the fact that there is no evidence of alternative translation initiations up to the C-terminal end, according to the ATGpr predictor software (data not shown). Furthermore, Abdullah and co-workers described a nonsense mutation in the *DYM* gene just one codon upstream of the 21 codon. They described a reduction in the expression of the mutated transcript by RT-PCR and suggested the acting of NMD system (Abdullah et al., 2020). In addition, null mutations such as this one in the *DYM* gene are associated with DMC syndrome (el Ghouzzi et al., 2003). The patient´s phenotype is totally compatible with this pathology, which confirms that the p(Ser21Ter) mutation is the genetic cause of this syndrome.

The reasons why the external NGS sequencing company classified this variant as of uncertain significance are unknown. It is true that they recommended a family segregation analysis in order to classify it as pathogenic, but according to ACMG recommendations (Richards et al., 2015), it would have been sufficient to have verified that there is no second translation start after exon 2 or a possible alternative splicing that would have overridden the action of the NMD system. This fact highlights the work of translational genetics laboratories since sometimes they are able to get where these companies do not. In this way, family segregation analyses, functional analyses of variants of uncertain significance or analyses to confirm isodisomies such as this case can be performed. Thanks to this second line of genetic research, it has been possible to provide the patient with a precise diagnosis, putting an end to a diagnostic odyssey that could have lasted for many more years.

### UPiD/UPhD mechanism

Further analysis of family segregation and microsatellite markers revealed that the homozygous condition of the mutation is due to mixed UPiD/UPhD of chromosome 18. As the centromere is within the isodisomic region, the hypothesis proposed for the occurrence of this event would be that during paternal meiosis II, one of the male gametes mistakenly received both chromatids of one of the chromosomes 18 (Eggermann et al., 2018); but one of these chromatids had previously undergone a cross-linking with the other chromosome 18. Therefore, at the moment of fertilization, the female gamete may have contributed with a third chromosome, originating a trisomy 18 that would finally be compensated by randomly eliminating an extra chromosome, which in this case would be the maternal one. This compensatory mechanism is called trisomic rescue (Liehr, 2022) and would be the major mode of UPD formation (Eggermann et al., 2018) rather than gamete complementation. This mechanism would explain the homozygosity of the mutation, as it is located in the region where isodisomy occurs.

The incidence of UPD worldwide is not clearly determined due to, among others, publishing or methodological bias (Liehr, 2022), but it has been estimated to be around 1:3,500 (Robinson, 2000) and/or 1:2,000 (Nakka et al., 2019). Not all chromosomes undergo isodisomy with the same frequency, with chromosome 18 being one of the least reported. As far as we are concerned, there are only two studies reporting an UPD in chromosome 18, one of them segmental (Kariminejad et al., 2011; Morgan et al., 2018). Furthermore, mixed UPiD-UPhD from paternal origin in any chromosome is a very rare event (Scuffins et al., 2021). This evidence, together with the fact that the general paternal UPD is much less common than maternal UPD (Nakka et al., 2019; Liehr, 2022), shows that the case presented here must be an extremely rare event. However, this fact is quite surprising when trisomies on chromosome 18 causing the well-known Edwards Syndrome, have an incidence of 1/6,000 to 1/8,000 live births. This discrepancy between the frequencies of trisomies and the very rare UPD events described for the same chromosome could be explained by considering that the cases of UPD in chromosome 18 are being underestimated. Chromosome 18 is the one with the lowest gene density (Nusbaum et al., 2005). This could be an important reason to explain why this trisomy is compatible with a full-term pregnancy and even with a few months of life. This assumption is supported by the fact that chromosome 13 is also one of the chromosomes with the lowest gene density (Dunham et al., 2004), which could also allow babies with a trisomy on this chromosome to be born, the so-called Patau Syndrome. The lower gene density in chromosome 18 also reduces the frequency of variants and the consequent occurrence of recessive diseases, which until now have been the only clue to discovering UPD cases. For this reason, it is possible that UPD cases on chromosome 18 may be underestimated, as most of them go unnoticed by the clinical eye at birth, while a trisomy is always diagnosed given its relevant symptomatology.

### Imprinting consequences of UPiD in chromosome 18

Consequences of UPiDs are well known such as disrupting imprinting (Robinson, 2000; Eggermann et al., 2018; Nakka et al., 2019; Benn, 2021), and they are closely intermingled with imprinting disorders (Liehr, 2014, 2022). For these reasons, we analyzed the possible imprinting consequences of the UPiD-UPhD in the proband and we discovered a described gene with paternal imprinting (Morison et al., 2005; Li et al., 2010; Edfeldt et al., 2014; Chen et al., 2015; Hsu et al., 2016; Nieser, 2016; Geneimprint, 2022), *ELOA3* -previously called *TCEB3C-* with possible and important clinical consequences. It is located in the isodysomic region of the patient, specifically in the same cytogenetic band as the *DYM* gene, 18q21, and it has a monoallelic tissue-specific expression. *ELOA3* was considered as a putative tumor suppressor gene (Edfeldt et al., 2014; Nieser, 2016) which is involved in small intestinal neuroendocrine tumors (SI-NETs) development (Edfeldt et al., 2014; Stålberg et al., 2016; Scarpa, 2019; Hofland et al., 2020). This gene encodes elongin A3 (Yamazaki et al., 2002), a transcription elongation factor, that forms a stable complex with elongin B and C, encoded by *ELOB* -previously called *TCEB2*- and *ELOC* -previously called *TCEB1*-, respectively. It has been described that *ELOB* and *ELOC* play an important role in tumors suppression by von Hippel–Lindau (Edfeldt et al., 2014), supporting the role of *ELOA3* as a tumor suppressor gene.

Frequent loss of one copy of chromosome 18 in primary tumors and metastases has been observed in SI-NETs (Andersson et al., 2009; Cunningham et al., 2011; D. H. Kim et al., 2008; Kulke et al., 2008; Kytölä et al., 2001; Löllgen et al., 2001; Stancu et al., 2003; Tonnies et al., 2001; Walsh et al., 2011; Wang et al., 2005). Edfeldt and co-workers identified only one copy of ELOA3 in most primary SI-NETs tumors (89%) as well (Edfeldt et al., 2014). Regarding the *ELOA3* gene, it would be reasonable to think that the tumors that lose one of the chromosomes 18 are those of maternal origin, since they are the only ones that are potentially expressed. This hypothesis is supported by the fact that these types of tumors are epigenetically deregulated, while genetically they remain very homogeneous (Banck et al., 2013; Karpathakis et al., 2016; Stålberg et al., 2016). In the case of our patient, having both copies of *ELOA3* of paternal origin, it is possible that there is no expression of elongin A3 and this fact can particularly affect the intestinal tissue, causing, in part, a similar situation to that of SI-NETs patients who have lost part of or all of chromosome 18. Unfortunately, this hypothesis could not be confirmed as it is not possible to biopsy the patient’s intestinal tissue. We also can not state that the loss of function of *ELOA3* gene is a determinant in SI-NETs development although a loss of heterozygosity exists on chromosome 18. Further studies are required to better understand the development mechanism of SI-NETs.

In any case, it seems advisable to carry out a genetic counseling for the patient to have revisions with the digestive specialist in order to prevent or mitigate this potential severe pathology. These results highlight the importance of characterizing the nature of the mutations in each individual patient to obtain an accurate diagnosis.

### Future treatments

Currently, as in almost all rare diseases, there are no targeted molecular therapies for DMC syndrome. However, increasingly, precise drugs are being designed to act specifically depending on the mutation that the patient has. In that sense, we already have read-through agents (Pranke et al., 2018) and inhibitors of the NMD system, such as gentamicin, amlexanox and escin, used in patients with cystic fibrosis with class I mutations (Lopes-Pacheco, 2020). It would be interesting to investigate whether this type of drugs could be recycled for use in other diseases with the same molecular problem as in the case here presented. Although it may be too late for our patient, it is possible that by administering this type of drugs from birth, the development of the pathology could be mitigated or delayed.

Similarly, epigenetic therapies have already proved their effectiveness in the oncological field and other pathologies such as infectious diseases, metabolic and cardiovascular disorders (Das et al., 2009; Dunn & Rao, 2017). As other authors note for Prader-Willi or Beckwith-Wiedemann syndromes (Y. Kim et al., 2019; Papulino et al., 2020), it is possible to reduce CpG island methylation on paternal and/or maternal allele to restore the gene expression (Bartolomei & Ferguson-Smith, 2011). Further studies are needed to determinate whether these epidrugs might become a valid chance for UPD cases with aberrations in imprinting regions.

### Genetic counseling

After the precision genetic diagnosis in this family, a good and accurate genetic counseling is also essential, especially when parents wish to have more children, as was the case with this family. In this case, the genetic counseling was radically different than if a classical autosomal recessive inheritance pattern had been found. The risk of having another child with DMC would be now equal to that of the general population, being well below the 25% that it would have been in the case of a recessive inheritance. In fact, the parents are currently expecting another baby and the pregnancy is proceeding normally.

In conclusion, it is worth highlighting the diagnostic odyssey that most patients with rare diseases and their families undergo, where they do not obtain a definitive diagnosis until 3–12 years after the appearance of the first symptoms. This odyssey rests in the fact that most UPDs are not detected unless there is a translational genetics laboratory supporting the national health system that decides to perform a family segregation test beforehand. In order to minimize these risks, it is desirable that NGS companies choose to perform trio exomes. This approach would make it possible to detect homozygous mutations that are due to UPD, making the most of these technologies and, ultimately, improving diagnosis so that it is faster and more precise.

## Data Availability

The datasets for this article are not publicly available due to concerns regarding participant/patient anonymity. Requests to access the datasets should be directed to the corresponding author.
