# Supplementary material for: Case Report: Precision genetic diagnosis in a case of Dyggve-Melchior-Clausen syndrome reveals paternal isodisomy and heterodisomy of chromosome 18 with imprinting clinical implications
Source: Front Genet. 2022 Nov 18;13:1005573. doi: 10.3389/fgene.2022.1005573 (PMC9716064; doi:10.3389/fgene.2022.1005573)
Supplement: Supplementary file 1 [file Table1.pdf]

Supplementary Table 1. ExonNIM design on a panel of 179 genes associated with bone dysplasias and mucopolysaccharidosis.

Gene	Cromosome	Number of amplicons	Total Bases	Base coverage	Bases no incluyed	Global coverage
B3GALT6	chr1	4	990	591	399	0.597
CDKN1C	chr11	4	1738	1076	662	0.619
HOXD13	chr2	6	1032	824	208	0.798
SHOX	chrX	10	924	747	177	0.808
SHH	chr7	10	1389	1197	192	0.862
IDUA	chr4	21	1962	1707	255	0.87
CHSY1	chr15	13	2409	2173	236	0.902
CA2	chr8	9	783	708	75	0.904
EIF2AK3	chr2	27	3351	3043	308	0.908
ARSB	chr5	14	1631	1489	142	0.913
ENPP1	chr6	31	2778	2544	234	0.916
KIF7	chr15	31	4032	3701	331	0.918
GALNS	chr16	14	1569	1449	120	0.924
NAGLU	chr17	14	2232	2063	169	0.924
HGSNAT	chr8	19	1908	1777	131	0.931
CHST14	chr15	7	1131	1057	74	0.935
CLCN7	chr16	34	2418	2261	157	0.935
B4GALT7	chr5	9	984	934	50	0.949
OBSL1	chr2	45	6112	5821	291	0.952
EVC	chr4	28	2979	2839	140	0.953
OSTM1	chr6	9	1005	962	43	0.957
TNFRSF11A	chr18	15	1851	1776	75	0.959
COL9A3	chr20	32	2055	1977	78	0.962
RPGRIP1L	chr16	41	3948	3810	138	0.965
FBLN1	chr22	23	2580	2497	83	0.968
MATN3	chr2	11	1461	1416	45	0.969
CEP290	chr12	80	7440	7218	222	0.97
IHH	chr2	9	1236	1199	37	0.97
SH3PXD2B	chr5	21	2736	2661	75	0.973
FLNB	chr3	63	7941	7753	188	0.976
GNAS	chr20	30	5930	5791	139	0.977
FAM20C	chr7	18	1755	1716	39	0.978
HSPG2	chr1	136	13176	12899	277	0.979
LRP5	chr11	45	4848	4757	91	0.981
CC2D2A	chr4	49	5074	4985	89	0.982
EVC2	chr4	34	3970	3907	63	0.984
TMEM67	chr8	38	3151	3101	50	0.984
PAPSS2	chr10	16	1860	1837	23	0.988

Gene	Cromosome	Number of amplicons	Total Bases	Base coverage	Bases no incluyed	Global coverage
RUNX2	chr6	13	1947	1923	24	0.988
MYCN	chr2	10	1395	1379	16	0.989
FBXW4	chr10	11	1239	1227	12	0.99
ATP6V0A2	chr12	24	2571	2547	24	0.991
PPIB	chr15	7	651	645	6	0.991
PTPN11	chr12	17	1782	1768	14	0.992
TBX3	chr12	18	2232	2221	11	0.995
WDR35	chr2	36	3546	3533	13	0.996
COL1A2	chr7	55	4101	4089	12	0.997
COL9A2	chr1	34	2070	2064	6	0.997
MMP2	chr16	19	1986	1980	6	0.997
TRIP11	chr14	50	5940	5920	20	0.997
FGFR3	chr4	29	2572	2567	5	0.998
LRP4	chr11	48	5718	5704	14	0.998
NIPBL	chr5	79	8649	8629	20	0.998
PCNT	chr21	94	10011	9986	25	0.998
TNFSF11	chr13	8	954	952	2	0.998
WNT7A	chr3	8	1050	1048	2	0.998
GLB1	chr3	18	2189	2185	4	0.998
FMN1	chr15	31	3591	3586	5	0.999
NOTCH2	chr1	54	7602	7595	7	0.999
GNPTAB	chr12	33	3771	3766	5	0.999
ACP5	chr19	9	978	978	0	1
ALPL	chr1	15	1657	1657	0	1
ANKH	chr5	13	1479	1479	0	1
ANO5	chr11	27	2790	2790	0	1
ARHGAP31	chr3	29	4335	4335	0	1
ARSE	chrX	13	1770	1770	0	1
BMP2	chr20	7	1191	1191	0	1
BMPR1B	chr4	13	1509	1509	0	1
CANT1	chr17	10	1206	1206	0	1
CASR	chr3	19	3391	3391	0	1
CDH3	chr16	22	2490	2490	0	1
CHST3	chr10	9	1440	1440	0	1
CLCN5	chrX	19	2556	2556	0	1
COL10A1	chr6	12	2043	2043	0	1
COL11A1	chr1	92	5574	5574	0	1
COL11A2	chr6	70	5478	5478	0	1
COL1A1	chr17	57	4395	4395	0	1
COL2A1	chr12	60	4464	4464	0	1
COL9A1	chr6	42	2838	2838	0	1

Gene	Cromosome	Number of amplicons	Total Bases	Base coverage	Bases no incluyed	Global coverage
RUNX2	chr6	13	1947	1923	24	0.988
MYCN	chr2	10	1395	1379	16	0.989
FBXW4	chr10	11	1239	1227	12	0.99
ATP6V0A2	chr12	24	2571	2547	24	0.991
PPIB	chr15	7	651	645	6	0.991
PTPN11	chr12	17	1782	1768	14	0.992
TBX3	chr12	18	2232	2221	11	0.995
WDR35	chr2	36	3546	3533	13	0.996
COL1A2	chr7	55	4101	4089	12	0.997
COL9A2	chr1	34	2070	2064	6	0.997
MMP2	chr16	19	1986	1980	6	0.997
TRIP11	chr14	50	5940	5920	20	0.997
FGFR3	chr4	29	2572	2567	5	0.998
LRP4	chr11	48	5718	5704	14	0.998
NIPBL	chr5	79	8649	8629	20	0.998
PCNT	chr21	94	10011	9986	25	0.998
TNFSF11	chr13	8	954	952	2	0.998
WNT7A	chr3	8	1050	1048	2	0.998
GLB1	chr3	18	2189	2185	4	0.998
FMN1	chr15	31	3591	3586	5	0.999
NOTCH2	chr1	54	7602	7595	7	0.999
GNPTAB	chr12	33	3771	3766	5	0.999
ACP5	chr19	9	978	978	0	1
ALPL	chr1	15	1657	1657	0	1
ANKH	chr5	13	1479	1479	0	1
ANO5	chr11	27	2790	2790	0	1
ARHGAP31	chr3	29	4335	4335	0	1
ARSE	chrX	13	1770	1770	0	1
BMP2	chr20	7	1191	1191	0	1
BMPR1B	chr4	13	1509	1509	0	1
CANT1	chr17	10	1206	1206	0	1
CASR	chr3	19	3391	3391	0	1
CDH3	chr16	22	2490	2490	0	1
CHST3	chr10	9	1440	1440	0	1
CLCN5	chrX	19	2556	2556	0	1
COL10A1	chr6	12	2043	2043	0	1
COL11A1	chr1	92	5574	5574	0	1
COL11A2	chr6	70	5478	5478	0	1
COL1A1	chr17	57	4395	4395	0	1
COL2A1	chr12	60	4464	4464	0	1
COL9A1	chr6	42	2838	2838	0	1

Gene	Cromosome	Number of amplicons	Total Bases	Base coverage	Bases no incluyed	Global coverage
[illegible]	[illegible]	[illegible]	[illegible]	[illegible]	[illegible]	[illegible]
MGP	chr12	5	387	387	0	1
MKS1	chr17	19	1730	1730	0	1
MMP13	chr11	14	1416	1416	0	1
MMP9	chr20	19	2124	2124	0	1
NEK1	chr4	44	3861	3861	0	1
NKX3-2	chr4	8	1002	1002	0	1
NOG	chr17	4	699	699	0	1
NPR2	chr9	31	3144	3144	0	1
NSDHL	chrX	8	1122	1122	0	1
PEX7	chr6	13	972	972	0	1
PHEX	chrX	24	2250	2250	0	1
PIGV	chr1	9	1482	1482	0	1
PITX1	chr5	8	945	945	0	1
PLOD2	chr3	29	2277	2277	0	1
PRKAR1A	chr17	12	1146	1146	0	1
PTH1R	chr3	20	1782	1782	0	1
PTHLH	chr12	6	961	961	0	1
PYCR1	chr17	11	1114	1114	0	1
RASGRP2	chr11	23	1830	1830	0	1
RECQL4	chr8	34	3627	3627	0	1
ROR2	chr9	20	2832	2832	0	1
SALL1	chr16	23	7222	7222	0	1
SALL4	chr20	19	3162	3162	0	1
SERPINH1	chr11	9	1257	1257	0	1
SLC25A12	chr2	21	2037	2037	0	1
SLC26A2	chr5	13	2220	2220	0	1
SLC34A3	chr9	18	1800	1800	0	1
SLC35D1	chr1	17	1068	1068	0	1
SLC39A13	chr11	11	1216	1216	0	1
SMARCAL1	chr2	24	2865	2865	0	1
SOST	chr17	6	642	642	0	1
SOX9	chr17	11	1530	1530	0	1
SP7	chr12	10	1296	1296	0	1
SULF1	chr8	26	2616	2616	0	1
TBCE	chr1	18	1584	1584	0	1
TBX15	chr1	11	1491	1491	0	1
TBX5	chr12	13	1944	1944	0	1
TBXAS1	chr7	17	1794	1794	0	1
TCIRG1	chr11	25	2558	2558	0	1
TCTN3	chr10	17	1824	1824	0	1

Gene	Cromosome	Number of amplicons	Total Bases	Base coverage	Bases no incluyed	Global coverage
TGFB1	chr19	11	1173	1173	0	1
THPO	chr3	8	2280	2280	0	1
TMEM216	chr11	5	500	500	0	1
TNFRSF11B	chr8	9	1206	1206	0	1
TP63	chr3	21	2216	2216	0	1
TREM2	chr6	7	693	693	0	1
TRPS1	chr8	23	3885	3885	0	1
TRPV4	chr12	22	2900	2900	0	1
TYROBP	chr19	5	386	386	0	1
WISP3	chr6	9	1167	1167	0	1
WNT3	chr17	10	1068	1068	0	1
WNT5A	chr3	9	1238	1238	0	1
ZMPSTE24	chr1	12	1428	1428	0	1
GNPTG	chr16	10	918	918	0	1
GNS	chr12	17	1659	1659	0	1
GUSB	chr7	17	1956	1956	0	1
IDS	chrX	15	1827	1827	0	1
SGSH	chr17	16	1509	1509	0	1
